# Does a preceding hand wash and drying time after surgical hand disinfection influence the efficacy of a propanol-based hand rub?

**DOI:** 10.1186/1471-2180-6-57

**Published:** 2006-06-22

**Authors:** Nils-Olaf Hübner, Günter Kampf, Philipp Kamp, Thomas Kohlmann, Axel Kramer

**Affiliations:** 1 Institute of Hygiene and Environmental Medicine, Ernst Moritz Arndt University, Walther-Rathenau-Str. 49a, 17489 Greifswald, Germany; 2 BODE Chemie GmbH & Co. KG, Scientific Affairs, Melanchthonstrasse 27, 22525 Hamburg, Germany; 3 Institute for Community Medicine, Ernst Moritz Arndt University, Walther-Rathenau-Str. 48, 17487 Greifswald, Germany

## Abstract

**Background:**

Recently, a propanol-based hand rub has been described to exceed the efficacy requirements of the European standard EN 12791 in only 1.5 min significantly. But the effect of a 1 min preceding hand wash and the effect of one additional minute for evaporation of the alcohol after its application on the efficacy after a 1.5 min application time has never been studied.

**Methods:**

We have investigated a propanol-based hand rub (Sterillium^®^, based on 45% propan-2-ol, 30% propan-1-ol and 0.2% mecetronium etilsulfate) in three variations: with (A) and without (B) a 1 min hand wash before the disinfection of 1.5 min with immediate sampling after the disinfection; and (C) without a hand wash before the disinfection but with sampling 1 min after the disinfection. The efficacy of the three variations was compared to the reference treatment of EN 12791. All experiments were performed in a Latin-square design with 20 volunteers. Pre- and post-values (immediate and after 3 hr) were obtained according to EN 12791.

**Results:**

The 3 min reference disinfection reduced resident hand bacteria on average by 1.8 log_10 _steps (immediate effect) and 1.4 log_10_-steps (sustained effect) respectively. Method A was equally effective as the reference (immediate efficacy: 1.5 log_10_-steps; sustained efficacy: 1.6 log_10_-steps). Method B seemed to be more effective (immediate efficacy: 2.3 log_10_-steps; sustained efficacy: 1.7 log_10_-steps). Method C revealed the best efficacy (immediate efficacy: 2.3 log_10_-steps; sustained efficacy: 2.0 log_10_-steps). A comparison of all three treatment variations and the reference treatment revealed a significant difference for the immediate efficacy (p = 0.026; Friedman test), but not for the sustained efficacy (p = 0.430). A post-hoc-test for the immediate efficacy indicated a significant difference between methods A and C (p < 0.05; Wilcoxon-Wilcox test). Hence, none of the treatment variations was significantly less effective than the reference treatment.

**Conclusion:**

An application of the propanol-based hand rub for 1.5 min after 1 min hand wash fulfills the efficacy requirements of EN 12791. The efficacy can be improved to some extent by omitting the preceding hand wash and by awaiting the evaporation of the alcohol which is clinical practice anyway. The preceding hand wash has the most negative effect on the immediate effect. Based on our data hands should not be routinely washed before the disinfection period unless there is a good reason for it such as visible soiling.

## Background

Alcohol-based hand rubs are recommended and widely used for surgical hand disinfection in many countries [1,2]. The main aim of the pre-operative treatment of hands is the reduction of resident skin bacteria to a minimum in order to reduce the risk of surgical site infections in case of perforated surgical gloves [3]. Alcohols are considered to have better antimicrobial efficacy and dermal tolerance which are two clear advantages in comparison to antimicrobial soaps [4,5]. The application time for alcohol-based surgical hand rubs has become shorter over the last decades, and there was increasing evidence that with well-formulated hand rubs an equivalent efficacy on the resident hand flora can be achieved at an application time of 1.5 min [6,7]. At the same time evidence was cumulating to suggest that a 1 min hand wash which is recommended and often performed before the disinfection period may reduce the efficacy of the alcohol to some extent [8]. But this effect has so far only been studied for a 3 min application time of the hand rubs. The influence of a preceding hand wash as well as of drying by evaporation of the hand rub on the efficacy of disinfection with an application time of 1.5 min is unknown. Therefore, these influences were studied by determination of the efficacy of a hand rub according to EN 12791 and compared with the 3 min reference treatment [9]. The test design of EN 12791 ensures that the treated hands are sampled immediately after the specific application time even if they are still wet with the hand rub. In clinical practice, however, it is recommended for healthcare workers to put on surgical gloves only onto dry hands due to a better skin tolerance and a lower risk for impairment of glove integrity [2,10]. Therefore, the test design of EN 12791 does not reflect the clinical practice of surgical hand disinfection in that particular point.

## Methods

### Products and application

The following preparations were used: propan-1-ol (60%, v/v) as reference alcohol of EN 12791, and Sterillium based on 45% (w/w) propan-2-ol, 30% (w/w) propan-1-ol, and 0.2% (w/w) mecetronium etilsulfate (Bode Chemie GmbH & Co. KG, Hamburg, Germany). Sterillium was applied to the hands for 1.5 min as described recently [6]. The reference alcohol was applied to the hands for 3 min according according to EN 12791 [9].

### Wash phase

The volunteers washed their hands with a non-medicated soap (sapo kalinus) as described by EN 12791. Thereafter hands were rinsed with running tap water and dried with a sterile paper towel.

### Determination of the pre-values and post-values

Sampling, cultivation and calculation of values were done according to EN 12791. Volunteers rubbed the phalanges of one hand (randomly selected) for one minute in TSB (pre-value) or TSB with addition of 3% Tween 80, 3% saponin, 0.1% histidine, and 0.1% cysteine as neutralizers (immediate effect after disinfection or drying), because these neutralizers are shown to be effective [11]. The other hand was gloved for 3 hours for the assessment of the sustained effect. After taking off the glove sampling was done in the same way as for the immediate effect. For each volunteer the logarithmic reduction factor (RF) was obtained as the difference between the log_10 _pre-value and log_10 _post values.

### Disinfection phase

Each of the 20 volunteers was treated with the reference product for 3 min as well as with Sterillium for 1.5 minutes. Between each product application, a rest period of at least one week elapsed in order to allow the reconstitution of normal skin flora.

### Design

We have investigated surgical hand disinfection with Sterillium in three variations: with (A) and without (B) a 1 min hand wash before the disinfection of 1.5 min with immediate sampling after the disinfection; and (C) without a hand wash before the disinfection but with sampling 1 min after the disinfection. All three applications were tested in a Latin-square design against the reference treatment.

### Statistics

A product is considered effective for surgical disinfection if the mean of the RF of both the immediate and sustained effect is not significantly lower than the corresponding mean RF of the reference treatment [9]. Differences in the immediate and sustained effects between the reference treatment and the three variations of product application were investigated by non-parametric Friedman tests, followed by the Wilcoxon-Wilcox procedure for pairwise comparisons. A p-value < 0.05 was chosen to indicate a significant difference.

## Results

The 3 min reference disinfection reduced resident hand bacteria on average by 1.8 log_10 _steps (immediate effect) and 1.4 log_10_-steps (sustained effect; Table 1) An application of the hand rub after a 1 min hand wash was equally effective (immediate efficacy: 1.5 log_10_-steps; sustained efficacy: 1.6 log_10_-steps). An application of 1.5 min of the hand rub without a 1 min hand wash was slightly more effective (immediate efficacy: 2.3 log_10_-steps; sustained efficacy: 1.7 log_10_-steps). Application of the hand rub for 1.5 min followed by 1 min drying time revealed the best efficacy (immediate efficacy: 2.3 log_10_-steps; sustained efficacy: 2.0 log_10_-steps). A comparison of all three treatment variations and the reference treatment revealed a significant difference for the immediate efficacy (p = 0.026; Friedman test), but no significant difference for the sustained efficacy (p = 0.430). A post-hoc-test of pairwise differences for the immediate efficacy revealed only that method C was significantly more effective than method A (p < 0.05; Wilcoxon-Wilcox test). None of the pairwise comparisons of methods A to C with the reference reached statistical significance.

## Discussion

For the first time it was demonstrated that the efficacy requirements of EN 12791 can be fulfilled with a propanol-based hand rub applied for 1.5 minute time with three different application procedures. The lowest efficacy was observed with a preceding 1 min hand wash, the highest efficacy was found without a preceding hand wash and with an additional drying time of 1 min after the disinfection period.

It has been shown before that a 1 min hand wash followed by drying hands with a paper towel has a significant effect on skin hydration which significantly increases for up to 9 min [8]. At the same time it was found that the efficacy of different types of alcohols is impaired by a hand wash to some extent [8]. The value of a hand wash before the disinfection period has been questioned recently in a draft European recommendation on surgical hand disinfection for two reasons [12]: First, a 1 min hand wash does not really reduce the number of skin bacteria to a relevant extent [4,13]. Second, it has been suggested in some studies that the efficacy of the alcohol-based hand rub is even reduced if hands are washed before disinfection [13,14]. The difference in efficacy of the same treatment was even more remarkable with and without a 2 min hand wash [15]. We can now add similar findings with an application time of 1.5 minutes.

Practical implications have to be adressed, too. The whole procedure of surgical hand disinfection is usually performed in the surgical theater block. If we assume that the surgeon enters the theater block with clean hands then there is no need to wash them before the disinfection period. If we assume that the surgeon enters the theater block with dirty hands then there is something wrong on another level. If we assume that hands have become dirty in the surgical theater block they should be washed immediately after soiling [1]. In that respect there is no reason to include a 1 min hand wash into the standard procedure of pre-operative treatments of hands especially since recent evidence points out some clear disadvantages of the hand wash.

The best efficacy was found without a hand wash and with additional drying time after the application of the hand rub. The immediate effect was only marginally better (0.03 log_10_-steps), the sustained effect improved somewhat more (0.3 log_10_-steps). It is certainly correct that the efficacy of a hand rub which is applied for a specific time such as 1.5 min should be assessed exactly after 1.5 min. At the same time the application by the healthcare workers will be different in the hospital as they have to await the evaporation of any residual alcohol from their hands before gloving. But the effect of the additional drying time has to our knowledge never been investigated with an alcohol-based hand rub although it has a clear practical relevance. We observed a trend towards a better overall efficacy if hands are allowed to dry for 1 min after the disinfection period. This finding was expected and can be explained by the additional and variable contact time of the alcohol with the skin. That is why the healthcare worker can expect that the "real efficacy" of an alcohol-based hand rub for surgical hand disinfection is to some extent better than measured according to EN 12791.

## Conclusion

An application of the propanol-based hand rub for 1.5 min after 1 min hand wash fulfills the efficacy requirements of EN 12791. The efficacy can be improved to some extent by omitting the preceding hand wash and by awaiting the evaporation of the alcohol which is clinical practice anyway. The preceding hand wash has the most negative effect on the immediate effect. Based on our data hands should not be routinely washed before the disinfection period unless there is a good reason for it such as visible soil.

## Competing interests

The second author is paid employee of Bode Chemie GmbH & Co. KG, Hamburg, Germany.

## Authors' contributions

GK and AK designed the study, NOH, GK, PK and TK analysed the data, NOH, GK and AK wrote the manuscript. All authors read and approved the final manuscript.

## Figures and Tables

**Table 1:** Mean reduction ± SD of the resident hand flora of 20 volunteers by reference treatment (60% propan-1-ol for 3 min) or a 1.5 min treatment with a propanol-based hand rub applied in three different modes; experiments according to EN 12791 in a Latin-square design.

Formulation	Method	Time (min) of			Immediate effect		Sustained effect	
		washing	disinfection	drying	RF	p-value*	RF	p-value*
Propan-1-ol	Reference disinfection	1	3	-	1.79 ± 0.98	0.026	1.42 ± 0.90	0.430
Sterillium	A	1	1.5	-	1.51 ± 0.97^§^		1.62 ± 0.93	
Sterillium	B	-	1.5	-	2.29 ± 1.02		1.70 ± 1.02	
Sterillium	C	-	1.5	1	2.32 ± 0.93^§^		2.03 ± 0.91	

*Comparison of all four treatments (Friedman test)

^§^Significant pairwise difference (p < 0.05) in Wilcoxon-Wilcox test
